# Profiling of Learners in Medical Schools as a Move Toward Precision Education: Protocol for a Scoping Review

**DOI:** 10.2196/41828

**Published:** 2022-10-28

**Authors:** Hira Salman, Leigh Powell, Laila Alsuwaidi, Bhavana Nair, Shakeel Ahmed Tegginmani, Jalal Mohamadeya, Nabil Zary

**Affiliations:** 1 Institute for Excellence in Health Professions Education Mohammed Bin Rashid University of Medicine and Health Sciences Dubai United Arab Emirates; 2 College of Medicine Mohammed Bin Rashid University of Medicine and Health Sciences Dubai United Arab Emirates; 3 Department of Student Services & Registration Mohammed Bin Rashid University of Medicine and Health Sciences Dubai United Arab Emirates; 4 Al Maktoum Medical Library Mohammed Bin Rashid University of Medicine and Health Sciences Dubai United Arab Emirates; 5 SMART Services Mohammed Bin Rashid University of Medicine and Health Sciences Dubai United Arab Emirates

**Keywords:** learners, medical students, medical undergraduates, medical graduates, profiling of learner, learner profile, medical education, medical universities, precision education, student record, graduate education, data extraction tool

## Abstract

**Background:**

Academic experiences seek to get the best out of learners, maximizing performance and developing the skills and competencies needed to foster lifelong learning. The more personalized and tailored the academic experience among learners, the better the outcome. Precision education is a novel approach to research and practice, which is concerned with identifying and tailoring education to the precise needs of the learner. An emerging area of precision education is using data to develop learner profiles for a better understanding of individual learners relative to the characteristics and competencies of lifelong learners.

**Objective:**

This scoping review aims to identify literature that reports on profiling learners within medical schools. Our review, as described in this paper, will describe the characteristics being measured, the methods and data sources used to generate profiles, and the resulting profiles that emerge. This review aims to provide guidance to those supporting medical school learners on the current state of learner profiling.

**Methods:**

This scoping review will use the Population, Concept, and Context framework, published by Joanna Briggs Institute, adhering to the Preferred Reporting Items for Systematic Reviews and Meta-Analyses Extension for Scoping Reviews guidelines. The search strategy was developed in collaboration with a library specialist. An initial search was conducted in PubMed, ERIC, Google Scholar, Cochrane, CINAHL, and SCOPUS. Data will be extracted, and 2 authors will undertake the screening procedure using the Preferred Reporting Items for Systematic reviews and Meta-Analyses Extension for Scoping Reviews checklist.

**Results:**

The database searches yielded 166 results, and title and abstract screening of 135 extracted articles is currently underway after eliminating 31 duplicates. We anticipate the scoping review to be completed in the first week of October 2022. The final scoping review will present the findings in a narrative and pictorial fashion**.**

**Conclusions:**

This review will help guide scholars looking to understand the current state of learner profiling within medical schools.

**International Registered Report Identifier (IRRID):**

PRR1-10.2196/41828

## Introduction

### Background

Academic experiences must seek to get the best out of learners, maximizing performance and developing the skills and competencies needed to foster lifelong learning [1,2]. Lifelong learning is a concept involving the development of human potential through a continuously supportive process, beginning with self-directed activities that stimulate and empower individuals to acquire all the knowledge, values, skills, and understanding they will need throughout their lifetimes and to apply them with confidence, creativity, and enjoyment in all roles, situations, and contexts [3-7]. The more personalized and tailored the academic experience is to learners, the better the outcome [8,9]. Precision education is a novel approach to research and practice, which is concerned with identifying and tailoring education to the precise needs of the learner [10]. The fuel that powers precision education is data [11]. An emerging area of precision education is using data to develop learner profiles for a better understanding of individual learners relative to the characteristics and competencies of lifelong learners [12,13]. This can include dimensions such as motivation, metacognition, reflection, confidence, and regulation [13,14].

### Learner Profiling

Recent studies have investigated how best to profile learners to understand their relationship with how they learn. For example, a study in Finland used latent profile analysis to develop learner profiles around learner epistemic beliefs [14]. An exploratory study in the United Kingdom used cluster analysis to develop learner groups, with further examination to explore the differences between these student profiles and the extent to which cultural background impacts these profiles [13]. 

Efforts to profile learners are varied and focus on profiling a variety of characteristics, such as conceptions of learning [14,15]; epistemic beliefs [14]; learning styles [16]; self-directed learning skills, attributes, and strategies [16-18]; recognition of prior learning [19]; learning difficulties [20]; motivation level [9,14,21]; lifelong learning trends [22]; digital readiness for collaborative learning [23]; cognitive, metacognitive, and motivational strategies [24]; educational backgrounds, students’ study performance, and orientations [14,15]; situational reaction tendencies [25]; and coping styles, neuroticism, openness to experience, and agreeableness [26].

Learning strategies, approaches to learning, and perceptions of the learning environment as they relate to academic success are some areas that have been the focus of numerous studies generating the profiles of students pursuing higher education [13,27-30]. However, the focus has not been specific to medical students. Consolidating knowledge on this topic is crucial to facilitate teaching and drive future research and educational methods aimed at improving lifelong medical learners' attributes, skills, and competencies and their impact on academic performance. Considering that Arksey and O'Malley [31] state that the goal of a scoping review is to create a summative map of existing evidence and identify research gaps, this protocol describes our approach to conducting a scoping review on the practice of learner profiling in medical schools.

### Aim and Objectives

This study aims to review the profiling of learners in medical schools. In addition, this review investigates distinct learner profiles based on abilities and characteristics of lifelong learning. Therefore, we intend to answer the following research questions: (1) How are learner profiles generated in medical schools? (2) What methodologies are used to characterize learners in medical schools? (3) What characteristics are being measured to develop learner profiles?

## Methods

### Key Considerations

This review will use the Joanna Briggs Institute’s Population, Concept, and Context (PCC) methodology [32] for scoping reviews to determine the research subjects’ suitability. In addition, the PRISMA-ScR (Preferred Reporting Items for Systematic Reviews and Meta-Analyses extension for Scoping Reviews) reporting guidelines and checklist [33] will be used at the reporting stage. Modifications of the approach, when needed, will be considered throughout the review process. The updated Joanna Briggs Institute (JBI) methodological guidelines for the conduct of scoping reviews [34] suggest breaking down the review process into 9 distinct steps, starting from defining and aligning the objectives and questions; developing and aligning the inclusion criteria with the objectives and questions; describing the planned approach to evidence searching, selection, data extraction, and presentation of the evidence; searching for the evidence; selecting the evidence; extracting the evidence; analysis of the evidence; presentation of the results; and summarizing the evidence in relation to the purpose of the review, making conclusions and noting any implications of the findings.

### Eligibility Criteria

#### Sample

Previously published studies with the target population of medical students will be included.

#### Concept

All articles included will relate to learning profiling in a medical school context. This includes the characteristics being measured, the methods and data sources used to generate profiles, and the components of the final profiles themselves.

#### Context

Our scoping review will only include studies conducted in medical schools.

#### Types of Sources

This scoping review will include peer reviewed articles whose primary focus is on profiling medical learners. We will not include letters, comments, conference abstracts, editorials, doctoral theses, systematic reviews, non–peer-reviewed articles, and gray literature. Studies published in a language other than English will not be included. Table 1 provides a summary of the inclusion and exclusion criteria.

**Table 1:** Inclusion and exclusion criteria.

Criterion	Inclusion	Exclusion
Study type	Peer-reviewed articles	Letters, comments, conference abstracts, editorials, doctoral thesis, non–peer-reviewed studies, systematic reviews, and gray literature
Time period	Any	N/A^a^
Participants	Medical students	Other health care students including physiotherapists, nurses, postgraduates, physicians, social sciences students, and students in other higher education departments
Focus area	Key competencies areas addressed, data sources, tools and instruments, and statistical analysis for profiling learners	Studies without data sources will be excluded
Outcome	Generated profiles based on attributes and learning in medical schools	N/A
Language	English	Non-English

^a^N/A: not applicable.

### Search Strategy

According to JBI, the bibliographic database search should be carried out in accordance with a step-by-step plan. In the first part of the limited search process, we looked through the PubMed and ERIC databases by analyzing the words in the titles and abstracts of the papers that have been retrieved, as well as an examination of the index keywords that have been used to characterize the publications. In the second step, we used all of the previously identified keywords and index terms to conduct a second search of the databases, including PubMed, ERIC, SCOPUS, Cochrane, CINAHL, and Google Scholar, to retrieve peer-reviewed research papers that are relevant to our objective. After screening, the final set of included studies were imported into the reference management software Zotero, where duplicates were removed. Coauthor ST, the information specialist, helped establish a comprehensive search strategy by ensuring that the search strings are inclusive, and the selected databases are relevant to our research. The used keywords and search strings are mentioned in Tables 2 and 3.

**Table 2:** Keywords and Medical Subject Headings (MeSH) terms for the Population, Concept and Context framework.

Population		Concept		Context	
MeSH	Keywords	MeSH	Keywords	MeSH	Keywords
Students, Medical	Medical student*		Learner profiling	Education, Medical, Undergraduate	Medical Education
	Learners		Profiling of learner*	Education, Medical	Medical School*
	Medical undergraduates		Learner profile*	Education, Medical, Graduate	

**Table 3:** Search strings.

Search strings	Database	Results, n
((((Students, Medical[MeSH Terms]) OR (Learners)) OR (Medical students)) OR (Medical undergraduates)) AND ((((Profiling of learner*) OR (Learner profile*) OR (learner profiling) AND ((((Education, Medical, Undergraduate[MeSH Terms]) OR (education, graduate[MeSH Terms])) OR (education, medical[MeSH Terms])) OR (Education, Medical, Graduate[MeSH Terms]))	PubMed	42
(“Medical student*”)) AND ((“ Profiling of learner”) OR (“Learner profile*”)) AND ((“Medical education”) OR (“Medical Universit*”))	Google Scholar	53
“Learner profile” AND “medical education”	ERIC	17
“Learner profile” AND “medical students” AND “Medical school”	CINAHL	19
“Learner profile” AND “medical students” AND “Medical school”	SCOPUS	28
“Learner profile” AND “medical students” AND “Medical school”	Cochrane	7

### Study Selection and Screening

The web-based software platform Rayyan [35] will streamline the screening process. As recommended by Levac et al [36], titles and abstracts will be screened separately by 2 reviewers (HS and LP) to ensure that they are relevant to the review. A discussion of article selections and search strategies will occur at various points throughout the screening process. It is possible to uncover and integrate new keywords, sources, and search phrases into the search strategy. The reviewers will go through the complete text and apply the inclusion criteria mentioned in Table 1 for papers that were not disqualified on the basis of the title or abstract. A single arbitrator (NZ) will determine any disagreements among reviewers.

Per the PRISMA-ScR statement [33], the final review will be delivered in narrative and pictorial form. In addition, the final research will include an appendix with information on the eliminated papers after full-text review and validation.

### Data Extraction

A data extraction tool will be developed to extract data from included publications. Textbox 1 displays the data to be extracted. The data extraction tool may be adjusted and amended during the process, and any changes will be documented in the final report. Relevant information on the population, topic, setting, research methodology, and significant results will be retrieved. Any disagreements between reviewers will be resolved through discussion or the involvement of an additional reviewer during the screening process [37]. Reviewers may also contact article authors to seek missing or extra information.

**Data extraction protocol.**

**Data to be extracted from articles**

All study information will be extracted and processed using a data charting table. The data to be extracted and explained include the following:

**General study information**

General study specifics entail bibliographic information, purpose or objective, study design, and participants.

**Related to profiling of learners**

1. We will collect the characteristics measured in the study.
2. What instruments or tools were used to measure the scales?
3. What data sources are used to generate learner profiles?
4. What kinds of learner profiles were generated?
5. We will look for any associations of generated profiles with study approaches, learning strategies, academic success, and study exhaustion.
6. Collect information regarding the various statistical procedures used for data analysis.

### Data Analysis

Abstracted information from all the included articles will be compiled, and the results will be presented independently by two researchers (HS and LP) to capture the extent of the literature. Tables of the extracted data will be developed to provide an overview of the information from each paper. This overview will be followed by a narrative presentation of the synthesized mapping of the included literature and descriptive qualitative content analysis to identify or clarify concepts or definitions within a field and highlight qualities associated with a concept. In addition, the research team will analyze findings regarding the study’s overall purpose and evaluate the implications for future research, practice, and policy.

## Results

**Figure 1:**
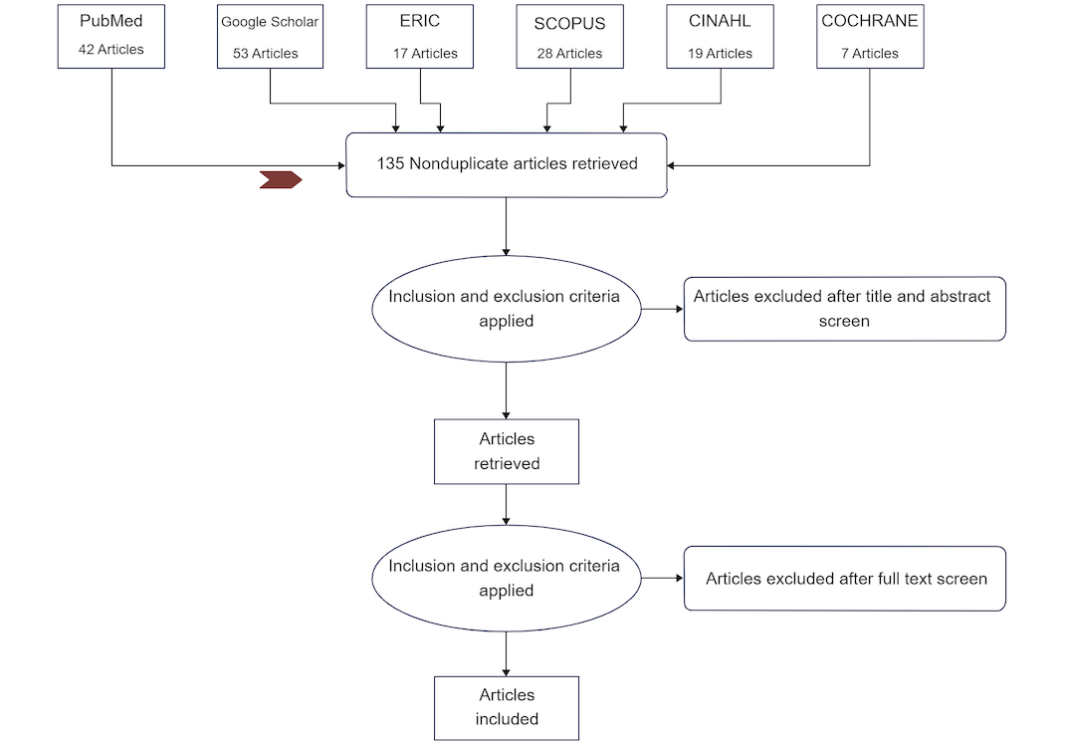
Flowchart of Preferred Reporting Items for Systematic Reviews and Meta-Analyses (PRISMA) for the scoping review procedure. Small colored arrow represents the current status.

Initial database searches revealed 166 studies. The database searches were completed in August 2022, and title and abstract screening of 135 extracted articles is currently underway after the elimination of 31 duplicates; we anticipate the final results in the first week of October 2022. The extracted findings will be presented in accordance with the scoping review’s goals and questions. In accordance with Peter et al [32], our findings will comprise 2 major components. The first component will include a PRISMA flowchart describing the research selection procedure (as shown in Figure 1). The most important data or findings pertinent to the scoping review’s goals or queries are presented in the second part. Upon evaluation of the contents of the included evidence, this could be further refined throughout the review phase. The outcomes of a scoping review will be displayed as a map of the data gathered from the studies that were included, both in tabular form and in a descriptive format that is in line with the review’s goals and scope. The components of the PCC inclusion criteria will help determine the most effective method for communicating the scoping review’s findings to the audience.

## Discussion

### Background

The essence of “precision” conveys the necessity of collecting complex and multidimensional data related to learners’ characteristics, behavior, interactions, and performance, enabling educators to better understand learners’ needs. Profiling learners from multiple perspectives and points of view can be a useful pathway to obtaining a good understanding. Learner profiling has been previously investigated in higher education; however, no review has been undertaken on learners in medical schools. This review will map the research in this field to better understand how learner profiles are being developed and are being used in medical education.

Profiling learners in medical schools can be a potentially helpful strategy for facilitating successful academic and lifelong learning outcomes [38]. By conducting this scoping review, we strive to harmonize distinct styles, strategies, and methods that might increase clarity in this domain. In addition, by reporting on the validated instruments, resources, and design and development strategies used to generate learner profiles in medical education, we will inform those seeking to develop learner profiles in their setting, potentially leading to an efficient, personalized learning process [39]. We intend to disseminate our results through publication in a peer-reviewed journal.

### Limitations

First, the review methodology does not include gray literature such as book chapters, theses, short papers, editorials, non–peer-reviewed reports, and conference abstracts; second, we will only include studies written in English owing to the feasibility and limitation of resources. To mitigate the publication biases, adhering to the JBI’s principles for scoping reviews will help to ensure a rigorous and logistical methodology in terms of the research framework, a search strategy including search strings and keywords, and searches of well-established academic databases. Lastly, it may not be possible to generate practice recommendations based on the outcomes of this scoping review since no evaluation of methodological quality or rating of evidence levels will be conducted. However, this study will identify further research gaps and the potential need for additional systematic reviews.

### Conclusions

This scoping review will provide a foundational understanding of the current state of profiling learners in medical schools. The outcome of this scoping review can help further the field of precision education and potentially promote lifelong learning to help produce learners who have the attributes necessary to fulfill their individual and collective responsibilities to society and who are prepared to serve the fundamental purposes of medicine. This scoping review protocol describes the design for the review on medical school learner profiling and outlines the methodological challenges and steps taken to ensure rationality. The latter can be applied and expanded on by researchers outside the medical field.

## Abbreviations

JBI: Joanna Briggs Institute
PCC: Population, Concept, and Context
PRISMA-ScR: Preferred Reporting Items for Systematic Reviews and Meta-Analyses Extension for Scoping Reviews

## Data Availability

All data relevant to the study will be provided in the scoping review.
